# Integrating the Genetic and Physical Maps of *Arabidopsis thaliana*: Identification of Mapped Alleles of Cloned Essential (*EMB*) Genes

**DOI:** 10.1371/journal.pone.0007386

**Published:** 2009-10-08

**Authors:** David Meinke, Colleen Sweeney, Rosanna Muralla

**Affiliations:** Department of Botany, Oklahoma State University, Stillwater, Oklahoma, United States of America; USDA-ARS/Donald Danforth Plant Science Center, United States of America

## Abstract

The classical genetic map of *Arabidopsis* includes more than 130 genes with an embryo-defective (*emb*) mutant phenotype. Many of these essential genes remain to be cloned. Hundreds of additional *EMB* genes have been cloned and catalogued (www.seedgenes.org) but not mapped. To facilitate *EMB* gene identification and assess the current level of saturation, we updated the classical map, compared the physical and genetic locations of mapped loci, and performed allelism tests between mapped (but not cloned) and cloned (but not mapped) *emb* mutants with similar chromosome locations. Two hundred pairwise combinations of genes located on chromosomes 1 and 5 were tested and more than 1100 total crosses were screened. Sixteen of 51 mapped *emb* mutants examined were found to be disrupted in a known *EMB* gene. Alleles of a wide range of published *EMB* genes (*YDA*, *GLA1, TIL1*, *AtASP38*, *AtDEK1, EMB506, DG1, OEP80*) were discovered. Two EMS mutants isolated 30 years ago, T-DNA mutants with complex insertion sites, and a mutant with an atypical, embryo-specific phenotype were resolved. The frequency of allelism encountered was consistent with past estimates of 500 to 1000 *EMB* loci. New *EMB* genes identified among mapped T-DNA insertion mutants included *CHC1*, which is required for chromatin remodeling, and *SHS1/AtBT1*, which encodes a plastidial nucleotide transporter similar to the maize Brittle1 protein required for normal endosperm development. Two classical genetic markers (*PY, ALB1*) were identified based on similar map locations of known genes required for thiamine (*THIC*) and chlorophyll (*PDE166*) biosynthesis. The alignment of genetic and physical maps presented here should facilitate the continued analysis of essential genes in *Arabidopsis* and further characterization of a broad spectrum of mutant phenotypes in a model plant.

## Introduction

Embryo-defective (*emb*) mutants are the most common class of mutants observed following chemical and insertional mutagenesis in *Arabidopsis*. Hundreds of mutants with a wide range of defects in seed development have been described [1]–[6]. Information on mutants disrupted in known genes is available at www.seedgenes.org. This database includes more than 350 genes and 600 mutant alleles [7]. A complete list of *emb* mutants disrupted in genes that remain to be identified is not available. An initial dataset of 250 *emb* mutants analyzed in our laboratory, including many that have not been cloned, was published 15 years ago [5]. Most of these mutants can be obtained through the *Arabidopsis* Biological Resource Center (ABRC) and can be found at TAIR, the central *Arabidopsis* database (www.arabidopsis.org). Another 1400 mutants derived from T-DNA insertion lines [6] but not yet cloned because they are either not tagged or unresolved with respect to tagging status are listed in a linked file at the SeedGenes website. Seeds from these Syngenta (Research Triangle Park) mutants can also be obtained from ABRC.

Because of their large numbers, embryo-defective mutants present a special challenge for saturation mutagenesis. With thousands of mutants available, determining which mutants are disrupted in the same gene can be overwhelming. Estimating the total number of *EMB* genes found throughout the genome is also difficult. We first began to approach these challenges 20 years ago by placing large numbers of *EMB* genes defined by mutation on the classical genetic map and then crossing heterozygotes segregating for mutations with similar map locations to test for allelism [8], [9]. The results were encouraging in several respects. First, we found 19 examples of duplicate alleles, including some with different phenotypes, among a collection of 110 mapped *EMB* genes. Second, we added a significant number of reliable genetic markers to the classical genetic map. Third, the frequency of duplicate alleles encountered within this limited sample suggested that *Arabidopsis* contained about 500 total *EMB* genes. This contrasted with an earlier estimate of 4000 *EMB* genes based on a comprehensive screen for mutants with defects in pattern formation [10]. A revised estimate of 500 to 1000 *EMB* genes was later obtained by examining the frequency of duplicate alleles within a large collection of cloned T-DNA mutants [6]. These results seemed to indicate that identifying all of the *EMB* genes might eventually become an attainable goal.

Several complementary strategies are being used to identify additional *EMB* genes in *Arabidopsis* [7]. Most large-scale efforts involve reverse genetic analysis of candidate genes that are either orthologs of essential genes in other organisms, fail to generate a knockout homozygote, or share a common cellular process, metabolic pathway, or protein interactor with a known *EMB* gene product. Other *EMB* genes are being uncovered gradually as investigators observe a seed phenotype in knockout heterozygotes of their favorite gene of interest. Saturating for essential genes in *Arabidopsis* is needed to complete the genome-wide collection of knockout mutants, determine which genes give a loss-of-function phenotype, compare the null phenotypes of different types of genes, provide insights into genes with unknown cellular functions, and reach the long-term goal of determining the biological role of every gene in a model plant.

One valuable resource of *EMB* genes that has received little attention in recent years is the large collection of mapped (but not cloned) mutants analyzed in our laboratory [9]. We reasoned that if the existing collection of known *EMB* genes defined by mutation is around 30% to 50% saturated as potentially indicated by recent estimates, then a similar percentage of mapped (but not cloned) mutants should be allelic to cloned (but not mapped) mutants. We describe here the results of a study focused on the two largest chromosomes (1 and 5) that pursued this approach in considerable detail. This work provided us with an opportunity to update the classical genetic map, integrate this map with the known physical map, uncover the identities of 16 mapped *EMB* genes not previously cloned, identify four new *EMB* genes from mapped mutants tagged with T-DNA, and provide further information on the total number of genes with a loss-of-function phenotype affecting seed development in *Arabidopsis*.

## Results

### Updating the classical genetic map

The first comprehensive genetic map of *Arabidopsis* was published 25 years ago by Koornneef *et al.* [11]. This map included 76 genes with mutant phenotypes that were assigned chromosomal locations based primarily on the analysis of F_2_ plants produced from self-pollination of heterozygotes. Other genes with mutant phenotypes were added to the map over the years. Precise gene orders were often left unresolved because map positions were typically determined by comparing two-point recombination frequencies rather than by analyzing definitive three-point backcrosses. The most recent version of the map published 10 years ago [12] contained 462 loci and 469 total cM. However, that map included 110 genes placed initially on the recombinant inbred map [13] and then transferred to the classical map after adjusting for differences in chromosome lengths. Because this manipulation introduced additional uncertainties, we decided to limit the updated map presented here to genes with mutant phenotypes mapped relative to each other. We then queried public databases to determine which mapped genes had been cloned and whether a second allele with a different name was unknowingly assigned a slightly different chromosomal location based on work in another laboratory. We also contacted investigators for updated information on specific mutants, added a few loci from recent work in our laboratory, removed several genes where the estimated genetic location conflicted with the known physical location, and excluded mutants for which seed stocks were no longer available.

Three hundred and thirty-five genes are included on the updated genetic map summarized in Table 1. Forty-one percent (136) of these exhibit a seed phenotype when disrupted by a loss-of-function mutation. The remaining 199 genes exhibit some other mutant phenotype. Almost 90% of these genes have been cloned. By contrast, only 32% of the mapped *EMB* genes have been cloned, even when results of the present study are included. Over 90 mapped *EMB* genes remain to be cloned, including 34 on the two chromosomes examined here in detail. These represent promising candidates for *EMB* genes not represented in the SeedGenes database. Table S1 lists 22 genes with other phenotypes that remain to be cloned. Twelve of these were included on the initial genetic map of Koornneef *et al*. [11].

**Table 1:** Updated classical genetic map of *Arabidopsis*.

	Loci on classical genetic map			Cloned genes on classical map			Mapped genes not cloned	
Chromosome	Total	*EMB*	Other	Total	*EMB* a	Other	*EMB*	Other
1	89	37	52	65	16 (6)	49	21	3
2	46	19	27	31	5 (1)	26	14	1
3	59	24	35	36	3	33	21	2
4	65	27	38	34	3 (2)	31	24	7
5	76	29	47	54	16 (11)	38	13	9
Total	335	136	199	220	43 (20)	177	93	22

a Numbers in parentheses indicate *EMB* genes identified in this report.

**Figure 1:**
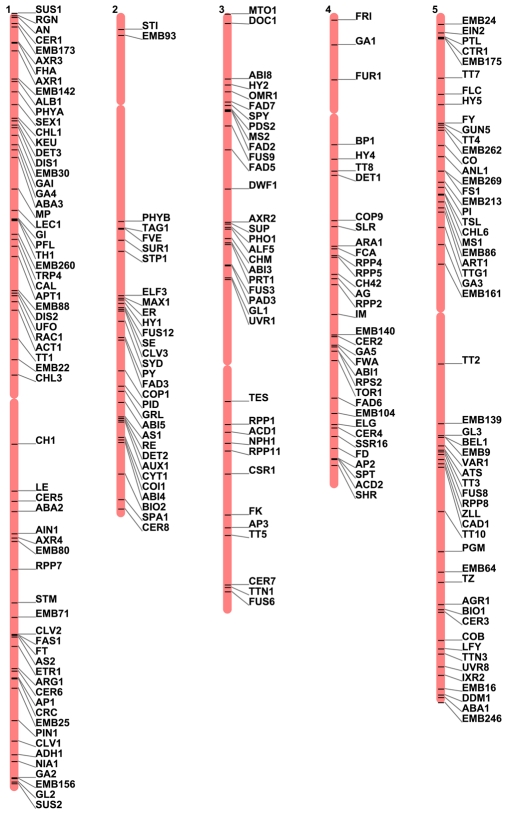
Placement of 220 cloned markers from the classical genetic map of *Arabidopsis* on the sequence-based physical map. Centromere locations are marked with constrictions. The map visualization tool was obtained from TAIR (www.arabidopsis.org/jsp/ChromosomeMap/tool.jsp).

A complete list of 335 genes included on the updated map, along with known locus numbers, is presented in Table S2. Two different versions are provided; one arranged by estimated genetic location and the other by known physical location. Many examples of incorrect gene orders on the genetic map can be found. Most of these are likely the result of estimating map locations based on two-point recombination data from different laboratories. Overall, the estimated gene orders presented on the original Koornneef map, where common populations were subjected to standard mapping experiments, are remarkably consistent with known physical locations. Figure 1 illustrates the chromosomal positions of 220 cloned loci included on the updated genetic map. The chromosome mapping tool used to visualize gene locations was obtained through TAIR. Most of the chromosomal regions devoid of mapped genes with a mutant phenotype are nevertheless populated by loci that were included six years ago in a comprehensive dataset of all known *Arabidopsis* genes with a mutant phenotype of any kind [14].

### Integrating the genetic and physical maps

We decided to update the genetic map and identify additional examples of mapped genes already cloned in order to estimate more precisely the physical locations of mapped *EMB* genes that remained to be cloned. This information was required before we could compile lists of cloned *emb* mutants that were logical candidates for allelism tests with mapped *emb* mutants localized to similar regions of the genome. First we needed to plot the estimated genetic position versus known physical location for all mapped and cloned genes regardless of mutant phenotype. We decided against using sequence coordinates to track physical distances because this value was cumbersome and not readily derived from the locus number. We also chose not to use locus numbers to measure physical distance, in part because this made it difficult to visualize the number of genes within a defined physical region. We devised instead a simple measure of physical distance, the estimated gene number (EGN), which was derived by subtracting 1000 from the locus number, because for chromosomes 1, 2, 3, and 5, locus numbers begin with 01010 and not 00010, and then dividing by ten because locus numbers for adjacent genes are typically separated by ten. The EGN for At1g02580 is therefore 158 (02580 – 01000 divided by 10). For chromosome 4, we simply dropped the final digit because locus numbers on that chromosome begin with 00020. Although the EGN is an imperfect measure of physical distance, it can be easily derived, readily visualized, and has minor inconsistencies when compared with genetic map distances. The utility of this measure of physical distance was also confirmed by the results described here.

**Figure 2:**
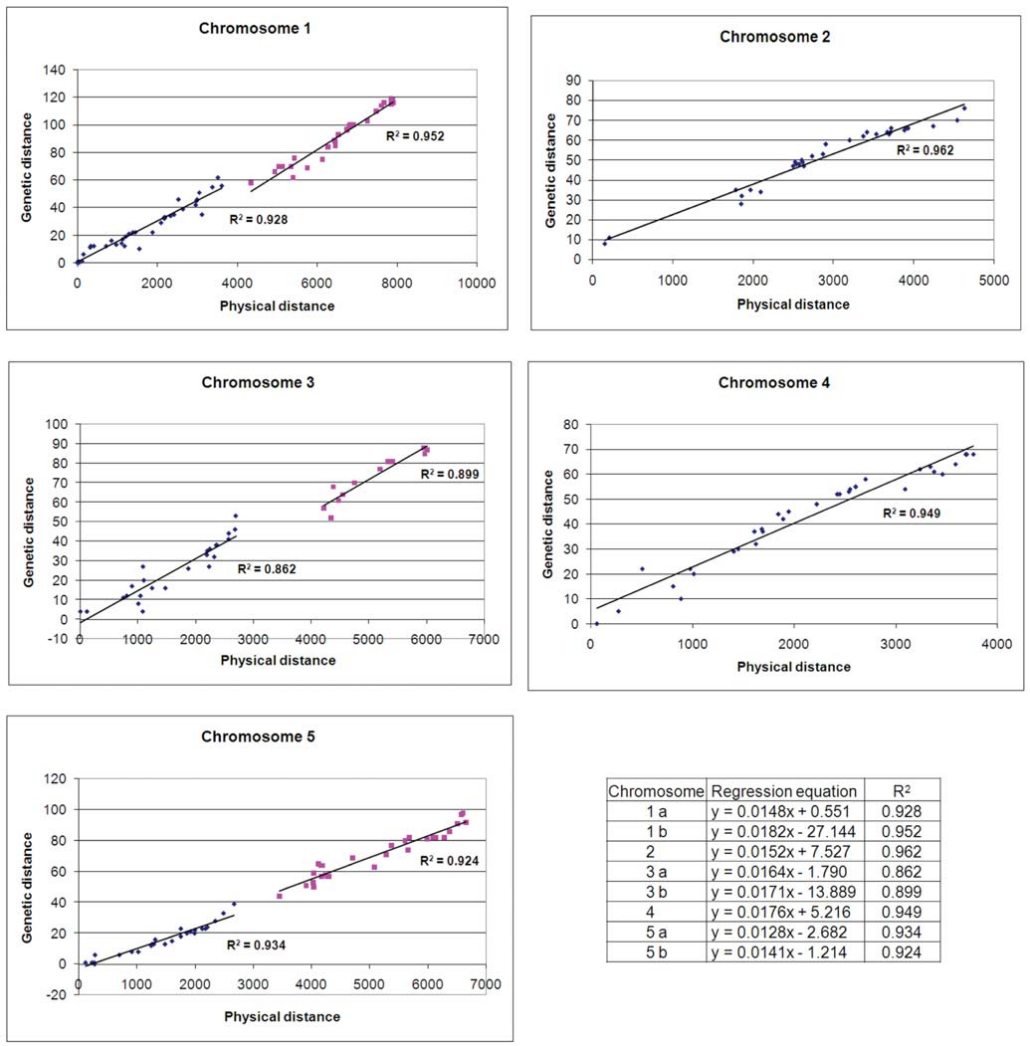
Comparison of genetic and physical distances for cloned markers on the classical genetic map of *Arabidopsis*. Genetic distances are measured in cM. Physical distances are measured in estimated gene numbers (EGN) as explained in the text. Linear regressions were calculated for each arm of chromosomes 1, 3, and 5 and for both arms combined of chromosomes 2 and 4. Equations used to generate each regression line and to compare genetic and physical distances chromosome-wide are noted.

Scatter plots showing the relationship between genetic and physical distances are shown in Figure 2. For chromosomes 1, 3, and 5, separate regression lines are presented for each chromosome arm in order to minimize differences in recombination rates across the centromere. A single regression line was used for chromosomes 2 and 4 because few genetic markers are located above the centromere. Similar regression values were obtained (e.g. R^2^ = 0.930 vs 0.928 and 0.959 vs 0.952, chromosome 1a, 1b; and 0.945 vs 0.934 and 0.921 vs 0.924, chromosome 5a, 5b) when physical distance was determined using the sequence coordinate midpoint (TAIR 7.0) of each mapped gene instead of EGN. These results provide further confirmation of the validity of the approach used here to estimate physical distance. Information about mapped genes flanking each centromere is presented in Table S3. The estimated location of each centromere was determined by averaging locus numbers flanking sequenced BACs rich in centromeric repeats. Five cloned loci with a genetic map location more than 15 cM removed from the expected location based on chromosome-wide regression lines were removed from the final version of the map. These loci are *PDS1* (At1g06570), *VAR2* (At2g30950), *FPA* (At2g43410), *TT6* (At3g51240), and *FAS2* (At5g64630). Placement of these loci on the genetic map appears to be inaccurate but the gene identities are correct. Another mapped locus (*CHL2*) with contradictory mapping information and a questionable gene assignment was also removed.

### Identifying candidates for allelism tests

The approach we took in the past when looking for duplicate alleles among mapped *emb* mutants was to limit complementation tests to mutants disrupted in genes that mapped within 5 cM of each other [9]. This strategy balanced inherent uncertainties about map locations with a desire to limit the total number of crosses performed. The project described here introduced another variable, correlating genetic position with physical location, and involved a greater number of crosses. Because we used estimated gene numbers to measure physical distances, we decided to count the total number of locus numbers assigned to each chromosome (TAIR version 7.0) and divide this number by the total number of cM per chromosome. The resulting genome-wide average [66 locus numbers (EGN) per cM] was fairly consistent across the five chromosomes (Table 2). We then established several priority levels for allelism tests based on map alignments. The first level (A) involved cloned *EMB* genes that were 200 EGN above or below the projected physical location of a mapped (but not cloned) *EMB* gene based on data presented in Figure 2. The second level (B) expanded the window to 300 EGN, slightly less than the estimated 5 cM window. A third level (C) combined the larger window and a single regression line based on data for the entire chromosome rather than separate lines for each chromosome arm. A fourth level (D) was limited to a few genes that seemed to be further apart but were still chosen for analysis.

**Table 2:** Relationship between locus numbers and cM.

Chromosome	Arm	Locus numbers^a^	cM	Loci/cM
**1**	**Both**	**8,000**	**122**	**66**
	a	3,945	57	69
	b	4,055	65	62
**2**	**Both**	**4,894**	**77**	**64**
	a	920	13	71
	b	3,974	64	62
**3**	**Both**	**6,297**	**96**	**66**
	a	3,755	54	70
	b	2,542	42	61
**4**	**Both**	**4,691**	**76**	**62**
	a	771	8	96
	b	3,920	68	58
**5**	**Both**	**6,896**	**98**	**70**
	a	3,005	42	72
	b	3,891	56	70
**Combined**	**Total**	**30,778**	**469**	**66**

a Based on TAIR 7.0 genome release (www.arabidopsis.org).

After identifying the cloned *EMB* genes that appeared to be located in the vicinity of each mapped *EMB*, we next asked whether the two mutant phenotypes appeared to be consistent with potential allelism. This was often difficult to resolve with confidence because the intragenic locations of mutations in mapped (but not cloned) *emb* mutants remained unknown. However, we usually excluded combinations that seemed unlikely, such as a cloned, putative null allele that exhibited a much later embryo phenotype than a mapped, untagged mutant. We also included some questionable combinations, especially when there were few other candidates available. Nevertheless, some of the mapped mutants that were tested but remain unresolved may be disrupted in a known *EMB* gene that was simply not chosen for crosses.

Approximately 200 sets of complementation tests and more than 1100 individual crosses involving pairwise combinations of candidate alleles were performed. Fifty-one mapped loci (27 chromosome 1; 24 chromosome 5) and 115 cloned loci (63 chromosome 1; 52 chromosome 5) were included. Crosses that failed to demonstrate allelism are summarized in Table 3. Additional details are provided in Table S4. Most of the 166 pairwise combinations that complemented involved genes estimated to be within the 300 EGN window described above (60% A; 27% B; 5% C; 8% D). Sixteen combinations that revealed allelism (failed to complement) and produced F_1_ siliques with the expected frequency of defective seeds are listed in Table S5. The distribution of these successful combinations among the four distance (window) classifications was almost identical to that found with crosses that complemented. In other words, crosses involving genes that seemed to be very close to each other based on predicted map alignments were not more likely to reveal allelism than those that appeared to be somewhat further apart, reflecting once again the inherent uncertainties associated with short distances. The frequency of allelism found (16 of 51 mapped loci with existing collection of 350 cloned *EMB* genes) suggested a level of saturation (30%) consistent with past estimates of 1000 total *EMB* genes [6].

**Table 3:** Cross combinations that complemented in allelism tests (different genes involved).

Chromosome	cM	Mapped *EMB* a	Gene identified^b^	Cloned genes found to be different from mapped locus indicated					
1a	3	EMB233*	No	*EMB2386*	*RRP4*	*EMB1687*	*EMB2781*	*EMB2394*	
	11	*EMB176*	No	*EMB2781*	*GRP23*				
	11	*EMB237*	No	*EMB2781*	*EMB3101*	*EMB3102*	*EMB2411*	*GRP23*	
	12	EMB142*	Yes	*EMB2784*	*EMB2411*	*EMB2004*			
	26	*EMB43*	No	*AtCCMH*	*HYD*				
	27	*EMB232*	No	*AtCCMH*	*EMB3104*	*EMB2719*			
	27	*EMB10*	No	*AtCCMH*	*EMB1507*				
	29	*EMB131*	No	*EMB2719*	*EMB1507*	*RSH*	*AtCAF2*		
	29	*TTN2*	No	*None*					
	34	*EMB260*	Yes	*TTN10*	*EMB1968*	*EMB2204*			
	42	EMB88*	Yes	*EMB2733*					
	49	*EMB126*	No	*EMB2279*	*HISN2*	*EMB2733*	*EMB3003*	*EMB2756*	
1b	66	*EMB27*	No	*EMB3105*	*EMB3106*	*TRAUCO*			
	69	*EMB118*	No	*EMB3105*	*EMB1273*	*EMB3106*	*TRAUCO*	*EMB3107*	
	71	EMB244*	No	*TRAUCO*	*EMB3107*				
	76	*EMB80*	Yes	*EMB1011*	*EMB1860*				
	76	*EMB128*	No	*EMB1275*	*EMB3107*	*EMB1011*	*EMB1860*		
	84	*EMB71*	Yes	*EMB1674*	*EMB1220*				
	86	*SUS3*	No	*EMB3108*	*EMB1220*	*SCO1*	*ILA*		
	92	*EMB41*	No	*YDA*	*EMB1688*	*KNF*			
	97	*EMBRG2*	No	*EMB2813*	*EMB2814*	*AtZr1*	*PFI*		
	99	EMB179*	No	*EMB1688*	*TWN3*				
	102	*EMBRG3*	No	*PFI*	*EMB2184*				
	110	*EMB54*	No	*EMB1793*	*EMB2810*	*EMB1473*	*EMB2217*		
	113	*EMB17*	No	*EMB1473*	*EMB2217*	*EMB1047*	*EMB3110*	*WIN1*	
	113	*EMB120*	No	*TWN3*	*TPS1*	*EMB1135*	*EMB1427*		
	115	*EMB156*	Yes	*EMB1473*	*EMB1135*	*EMB2217*	*EMB2753*		
5a	1	*EMB24*	Yes	*EMB2730*	*EMB2771*	*EMB2735*			
	6	EMB256*	No	*EMB2771*	*EMB2789*	*EMB2735*	*PEX1*	*HISN6A*	*EMB3010*
	9	*EMB163*	No	*EMB1873*	*EMB2107*	*PAS2*			
	9	*EMB68*	No	*PEX1*	*HISN6A*	*EMB3010*	*EMB3135*	*EMB3011*	*CYL1*
	16	EMB262*	Yes	*None*					
	18	*EMB269*	Yes	*EMB2247*	*EMB3007*	*OEP80*			
	20	*EMB213*	Yes	*EMB3138*	*EMB2247*	*EMB3007*	*EMB1379*		
	23	EMB86*	Yes	*None*					
	27	*EMB2*	No	*EMB1379*	*CYL2*	*EMB1705*	*EMB1265*	*EMB3009*	*TAD3*
	30	*EMB108*	No	*EMB1705*	*EMB1265*	*EMB3009*	*TAD3*	*EMB2473*	*EMB2775*
	31	*EMB222*	No	*EMB1030*	*EMB1138*	*EMB1644*			
	39	EMB161*	Yes	*EMB2473*					
	40	EMB215*	No	*EMB1644*	*EMB3139*				
5b	51	*EMB139*	Yes	*EMB2744*	*EMB3008*	*GLA1*			
	56	*EMB141*	No	*EMB3008*	*EMB3012*	*GLA1*	*EMB2815*		
	59	*EMB9*	Yes	*EMB3012*	*EMB1276*				
	60	*EMB170*	No	*EMB1276*					
	71	*EMB64*	Yes	*None*					
	79	*EMB67*	No	*EMB2755*	*EMB3143*	*ZEU1*	*EMB2407*		
	82	*EMB209*	No	*EMB2731*	*EMB2755*	*EMB3143*	*EMB2407*		
	85	EMB87*	No	*EMB1629*	*ZEU1*	*DOM1*	*EMB2759*	*EMB2746*	*EMB2780*
	85	*EMB15*	No	*EMB1629*	*ZEU1*	*DOM1*	*EMB2759*	*EMB2746*	*EMB2780*
	91	*EMB16*	Yes	*DOM1*	*EMB2759*	*EMB2746*	*EMB2780*		
	92	*EMB246*	Yes	*EMB1692*	*EMB2759*	*EMB2746*	*EMB2036*		

a T-DNA tagged mutants are indicated with an asterisk.

b Yes represents mapped mutants disrupted in gene identified in this report.

### *EMB* gene identities revealed through allelism tests

The identities of 16 mapped *EMB* genes as revealed through genetic complementation tests are presented in Table 4. Previous work on these mutants is documented in publications extending over the past 30 years. Two of these mutants (*emb9* and *emb16*) were part of the initial collection of embryo lethals described in the 1970s by Meinke and Sussex [2]. These mutants were also included in a project that examined the distribution of aborted seeds in heterozygous siliques as a measure of gametophytic expression of *EMB* genes [15]. Another mutant (*emb64*) was identified from a collection of X-irradiated seeds generated in the 1980s by Joseph Ecker [5]. Three other EMS mutants (*emb24*, *emb36*, *emb71*) identified about the same time were part of a widespread effort to characterize the response of mutant embryos in culture [3], [16]–[18]. The remaining mutants were identified through forward genetic screens of T-DNA insertion lines generated by Ken Feldmann [4], [5], [19]. Some of these were shown through genetic analysis to be not tagged with T-DNA. One mutant with a late phenotype was also chosen for additional characterization at the seedling stage [20]. Three mapped loci (*EMB156, EMB86, EMB161*) were shown before [9] to be represented by duplicate mutant alleles. The remaining 13 loci were thought to be represented by a single mutant allele each.

**Table 4:** Mapped *EMB* gene identities revealed through allelism tests and TAIL-PCR.

Locus number	Identity revealed	Mapped locus^a^	Mutagen	Mapped alleles	References	Cloned locus	Known alleles^b^	References	Identity confirmed^c^	Predicted protein function
At1g08260	Cross; PCR	EMB142*	T-DNA	1	[4], [5], [9]	*EMB2284; EMB529; TIL1*	6	[21], [22]	Yes	DNA polymerase subunit
At1g24340	Cross	*EMB260*	T-DNA	1	[5]	*EMB2421*	1		No	Monooxygenase
At1g30610	Cross	EMB88* (T-1002)*	T-DNA	1	[4], [5], [9], [19], [36]	*EMB2279*	3		Yes	PPR protein
At1g55350	Cross	*EMB80 (T-98)*	T-DNA	1	[5], [9], [19]	*EMB1275; AtDEK1*	5	[23], [24]	Yes	Cysteine protease
At1g63700	Cross	*EMB71*	EMS	1	[5], [9]	*YDA*	2	[25]-[27]	Yes	MAP3K protein kinase
At1g79560	Cross	*EMB156; EMB36*	T-DNA; EMS	2	[5], [9], [18]	*EMB1047*	2		Yes	Chloroplast FtsH-like protease
At2g03050	PCR	EMB93* (T-562)*	T-DNA	1	[4], [5], [9], [19]	No Symbol	0		No	Mitochondrial transcription termination factor
At4g24270	PCR	EMB140*	T-DNA	1	[4], [5], [9]	No Symbol	0		No	RNA recognition motif
At4g32400	PCR	EMB104* EMB42	T-DNA; EMS	2	[4], [5], [9], [18]	*SHS1*	0	[28]	Yes	Plastid nucleotide export
At5g02190	Cross	*EMB24 (109F-1C)*	EMS	1	[3], [5], [9], [16], [17]	*AtAsp38*	1	[29]	Yes	Aspartic protease
At5g14170	PCR	EMB262*	T-DNA	1	[4], [5], [9]	*CHC1*	0	[30]	No	Chromatin remodeling
At5g18570	Cross	*EMB269*	T-DNA	1	[5]	*EMB3138*	1		Yes	GTP binding protein
At5g19620	Cross	*EMB213*	T-DNA	1	[5]	*OEP80*	2	[31]	Yes	Chloroplast envelope protein
At5g22800	Cross; PCR	EMB86* EMB263	T-DNA	2	[4], [5], [9], [19], [20]	*EMB1030*	2	[32]	Yes	Alanyl tRNA synthetase
At5g27740	Cross; PCR	EMB161* EMB251*	T-DNA	2	[5], [9]	*EMB2775*	2		Yes	Replication factor
At5g40160	Cross	*EMB139*	T-DNA	1	[5], [9]	*EMB506*	1	[33]	Yes	Ankyrin repeat protein
At5g41480	Cross	*EMB9 (79A)*	EMS	1	[2], [5], [9], [15], [17]	*GLA1*	2	[34]	Yes	Folate biosynthesis
At5g53860	Cross	*EMB64*	X-Ray	1	[5], [9]	*EMB2737*	2		Yes	Unknown
At5g66055	Cross	*EMB16 (123B)*	EMS	1	[2], [5], [9], [15]-[17]	*EMB2036; AKR*	2	[33]	Yes	Ankyrin repeat protein
At5g67570	Cross	*EMB246*	T-DNA	1	[5]	*EMB1408; DG1*	1	[35]	Yes	PPR protein

a Alias symbols noted in parentheses; T-DNA tagged mutants indicated with an asterisk.

b Already included at www.seedgenes.org when this project began.

c Phenotype already shown to be caused by disruption of locus indicated, usually through analysis of multiple insertion alleles.

Most of the cloned gene identities presented in Table 4 were confirmed before we started this project, either through molecular complementation or the analysis of multiple insertion alleles. The protein products appear to function in a variety of cellular processes, from DNA replication (*EMB2284*, *EMB2775*) and translation (*EMB1030*) to signal transduction (*YDA*), folate biosynthesis (*GLA1*), and chloroplast protein import (*OEP80, EMB1047*). Ankyrin repeat proteins (*EMB506, AKR*), PPR proteins (*EMB2279, DG1*), cysteine and aspartic proteases (*AtDEK1, AtASP38*), and proteins with uncertain cellular functions (*EMB2421*, *EMB2737*) are also included. Duplicate mutant alleles for 11 of these cloned genes have been isolated and characterized in considerable detail [21]–[35]. A simple allelism test was therefore sufficient in many cases to establish gene identities and resolve longstanding questions about mapped *EMB* loci.

One mutant studied 20 years ago (*emb24*) was particularly intriguing because mutant embryos cultured on a basal medium developed into viable plants that produced 100% defective seeds following self pollination [17]. All normal seeds were obtained when rescued homozygotes were crossed with pollen from wild-type plants. We reasoned at the time that *EMB24* likely performed an embryo-specific function. This hypothesis has now been confirmed by demonstrating that *emb24* is disrupted in *AtASP38*, which encodes an embryo-specific aspartic protease that has been postulated to limit programmed cell death during reproductive development [29]. The *emb24* allele exhibits a consistent seed phenotype (linear cotyledon stage embryo) and a simple inheritance pattern (no gametophytic lethality; 24.6% mutant seeds in siliques of selfed heterozygotes, 1020 total seeds screened; all normal seeds when used as a female in crosses with non-allelic *emb* heterozygotes, 385 total seeds screened). In contrast, the *AtAsp38* allele exhibits a combination of embryonic and gametophytic lethality, a wide range of seed phenotypes, and a complex inheritance pattern. The new allele described here appears to have more residual function than the original *AtAsp38* knockout, which may be useful in future efforts to understand the precise role of this gene in plant growth and development.

Another example of an informative allele identified here is a tagged T-DNA mutant (*emb88*) that was problematic in the past [36] because the rearranged insertion site included chromosome 5 sequences flanking a T-DNA insert located within an unknown *EMB* gene positioned on chromosome 1. This gene has now been identified through allelism tests as *EMB2279*, which encodes a pentatricopeptide repeat (PPR) protein required for normal embryo development. The abundance of these putative RNA binding proteins among collections of cloned *EMB* genes has been noted before [37], [38]. It seems fitting that the individual who characterized the *emb88* chromosomal rearrangement [36] also published a report on the diversity of essential PPR proteins in *Arabidopsis* [39].

**Figure 3:**
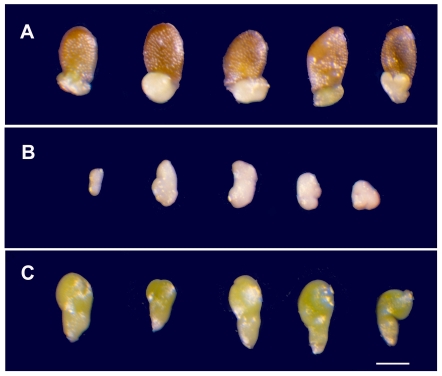
Terminal seed and embryo phenotypes of the *emb71* allele of *yoda*. (A) Mutant seeds exhibiting the external embryo phenotype at maturity. (B) Arrested embryos removed from the basal region of mutant seeds similar to those pictured above. (C) Second phenotypic class of *emb71* mutant embryos with a distorted cotyledon phenotype prior to desiccation. The two phenotypic classes combined account for 25% of total seeds produced from selfed heterozygotes. Scale bar = 220 µm.

The mapped mutant with the most unusual phenotype (*emb71*), described before as external [5] because some mutant embryos at maturity protrude from the base of the seed, was found to be disrupted in *YODA*, a gene that has received considerable attention in recent years. This locus encodes a component of a MAP kinase cascade that promotes proliferation of the basal cell lineage (suspensor) during embryo development [25] in addition to regulating stomatal spacing in the leaf epidermis [26]. Expression of *YODA* in the zygote is activated by the product of another gene (*SSP*) with an unusual parent-of-origin effect in which a transcript produced transiently in mature pollen is not translated until after fertilization [27]. The *emb71* allele identified here increases the diversity of *yoda* loss-of-function mutants available. The dramatic seed phenotype is shown in Figure 3. Terminal phenotypes were more readily characterized in the Columbia accession (*emb71*), where wild-type seeds are consistent in size and shape, than in the Landsberg (*erecta*) accession (*yda-1*; *yda-2*). The *yda-1* allele obtained from ABRC (CS6392) also segregated for a second (unlinked) *emb* mutation with a white seed phenotype. Two classes of mutant seeds were identified in siliques of *emb71* heterozygotes. One was dark green, often escaped detection unless seeds were dissected, and contained a distorted linear embryo with rounded cotyledons and a reduced hypocotyl. The second class was composed of pale yellow seeds of varying sizes and a disorganized embryo positioned at the base of the seed. This class gave rise to the external embryo phenotype later in development. Both classes were observed in F_1_ siliques produced from genetic complementation tests. We could not determine whether the external embryo phenotype resulted from unusual expansion of a developing embryo stuck in the micropylar region of the seed without a normal suspensor or from inappropriate activation of a degradation pathway within the seed coat.

### Molecular analysis of mapped T-DNA insertion mutants

Thermal asymmetric interlaced (TAIL) PCR was used to recover plant sequences flanking T-DNA inserts in several mapped *EMB* genes not identified through complementation tests. This work initially focused on chromosomes 1 and 5 but later expanded genome-wide. In several cases, TAIL-PCR results provided clues to the identity of a mapped locus scheduled for future crosses. Primers based on T-DNA border sequences were used in combination with different sets of degenerate primers that yielded informative products with insertion lines in the past [6], [40]–[42]. A full list of primer pairs tested is presented in Table S6. TAIL-PCR was ultimately pursued with a total of 21 tagged mutants from the Feldmann collection [4], [5]. These mutants are noted with an asterisk in Table S2. Informative PCR products were recovered from seven mapped mutants, including four that identified new *EMB* genes. These results are included in Table 4. Gene-specific primers were used in combination with T-DNA border sequences to confirm insertion sites. Information on flanking sequences obtained is summarized in Table S7. The overall success rate (7 of 21 tagged mutants resolved) was consistent with past experiences involving these insertion lines, which exhibit frequent chromosomal aberrations and a variety of truncated, duplicated, and rearranged insertion sites [4], [36]. We focused on those *EMB* genes that could be most readily identified with this approach.

Results obtained with *emb86* were particularly interesting because two different TAIL products were confirmed from chromosome 5. One identified the *EMB1030* locus (At5g22800) responsible for the mutant phenotype; the other revealed a linked locus (At5g20935) at the expected distance inferred from genetic analysis [19]. We demonstrated through reverse genetics that knockouts of that second locus (SALK_050034; SAIL_874_A12) did not exhibit a seed phenotype. This confirmed that the wild-type (*EMB/EMB*) transplants observed following selection on kanamycin [19] were the result of recombination between a tagged *EMB* locus (At5g22800) and a linked insert in another gene (At5g20935) not required for seed development.

Two *EMB* genes identified among mapped insertion lines were of particular interest because weak alleles with post-embryonic phenotypes had been described before [28], [30]. The first (*EMB104; SHS1; AtBT1*) involved two *emb* mutants with similar map locations that were known to be allelic [9]. One mutant was tagged with T-DNA (*emb104-1*); the other (*emb104-2*; *emb42*) was generated with EMS. We used TAIL-PCR to recover flanking sequence from *emb104-1* and direct PCR to confirm that the insert was located in the third (last) exon of At4g32420, which encodes a nucleotide transport protein. Another T-DNA mutant (*shs1*) with an upstream insertion site exhibits a salt-hypersensitive, ABA-insensitive phenotype that was at first attributed to a complete loss of function of a protein thought to be found in the endoplasmic reticulum [28]. However, these defects were later shown to result from reduced levels of a protein (AtBT1) localized instead to the inner plastid membrane, where it functions to export adenylates (AMP, ADP, ATP) into the cytosol [43]. We found a seed phenotype in two additional T-DNA insertion lines disrupted in At4g32420 (SALK_078665; GABI_785E11) and confirmed through allelism tests that both were allelic to *emb104*. The essential nature of this gene has therefore been confirmed. Terminal phenotypes of the SALK and GABI mutants were similar; arrested embryos were pale and ranged from the globular to elongate (triangular) stages of development. Giant suspensors and twin embryos were occasionally found. The phenotypes of both mapped alleles included distorted embryos at more advanced (linear cotyledon) stages of development, consistent with a somewhat higher level of normal gene function. Some of these embryos featured single or multiple cotyledon initials.

Another tagged mutant (*emb262*) contained a T-DNA insert localized within the 5′UTR of a gene (*CHC1*; At5g14710) known to play an important role in chromatin remodeling [30]. RNAi lines with reduced levels of CHC1 have been shown before to exhibit a severe dwarf phenotype and resistance to *Agrobacterium*-mediated root transformation [30]. We could not confirm through allelism tests that disruption of At5g14710 was responsible for the *emb262* seed phenotype because additional insertion lines were not available and plants homozygous for the RNAi construct did not exhibit a consistent defect in seed development. We suspect that the 35S promoter controlling RNAi expression was not active at a level sufficient to inhibit embryo development. Perfect linkage was nevertheless found between a kanamycin resistance marker and the mutant phenotype (0 *EMB/EMB* plants among 204 resistant transplants screened), consistent with T-DNA tagging [4]. We designate such mutants as “not confirmed” in the SeedGenes database, which indicates that gene identities revealed through TAIL-PCR are likely to be correct but remain to be proven. The *emb262* phenotype is somewhat unusual in that mutant embryos, which reach the cotyledon stage of development, are paler than expected based on the appearance of mutant seeds. Whether these embryos can be rescued in culture and produce plants that are resistant to *Agrobacterium*-mediated root transformation remains to be determined.

## Discussion

One long-term goal of research with the model plant, *Arabidopsis thaliana*, is to determine the biological consequences of disrupting each protein function and coding region of the genome. In principle, this can be accomplished through a combination of forward and reverse genetics and by constructing multiple mutants for redundant genes predicted to encode proteins with overlapping functions. A second goal is to determine what gene is disrupted in each mutant already characterized in detail. We describe here a comprehensive effort to address that second objective in relation to embryo-defective mutants, which constitute the most abundant class of mutants identified in *Arabidopsis*. Our results demonstrate that the altered gene in mapped mutants can often be revealed through genetic complementation tests with mutants disrupted in cloned genes localized to similar chromosomal regions. The method we present for integrating the classical genetic and physical maps of *Arabidopsis* is simple and robust, enabling rapid identification of candidate alleles.

One future application of this work may be found in associating classical genetic loci that remain to be cloned with genes examined through reverse genetics that exhibit similar loss-of-function phenotypes. We encountered one such example while updating the genetic map. The *THIC* gene of *Arabidopsis*, recently found to play an important role in thiamine biosynthesis [44], [45], appears based on map location and distinctive mutant phenotype to represent the classical *PY* locus, first described by Feenstra [46] and Rédei [47] more than 40 years ago. This association was not mentioned in either of the recent reports. We pursued another locus of interest (*ALB1*) after completing the map alignments shown in Figure 2. We reasoned that this albino seedling mutant might be allelic to an embryo-defective (*emb*) or pigment-defective embryo (*pde*) mutant in the SeedGenes collection. By crossing *alb1* heterozygotes to four potential alleles *(pde166, pde247, emb2784*, *emb2004*) based on similar map locations, we showed that *ALB1* is identical to *PDE166*, which encodes the CHLD subunit of the Mg-chelatase enzyme involved in chlorophyll biosynthesis [48]. The *ALB1* gene has therefore been identified.

The original *alb1* mutant allele (V157) was obtained from a collection of chlorina (yellow-green rosette) mutants generated by Robbelen starting in the 1950s [49]. This locus was later shown to be linked to the *angustifolia* genetic marker [50] on chromosome 1 [11]. The chlorina phenotype likely results from a partial loss of normal gene function. The *alb1* allele we examined (ABRC stock CS26), which appears to define the null phenotype, was first used by Van der Veen and colleagues [51] to study double reduction (chromatid segregation) in tetraploid plants. This locus was later chosen as a cell-autonomous marker to construct a fate map of the shoot apical meristem of mature seeds [52], [53] and to measure genomic instability in radiation-sensitive (*uvh1*) plants [54]. Two *pde166* (*alb1*) insertion alleles from the Syngenta collection [6] are included in the SeedGenes database. These CHLD null alleles, in combination with several CHLI mutants (*ch-42*; *cs*; *chli2*) with milder defects [55], should provide insights into the functions of specific Mg-chelatase subunits in chlorophyll biosynthesis.

We did not attempt here to document localized differences in recombination rates across the entire genome. This has been addressed before in a number of comprehensive studies involving humans [56] and several model organisms [57]. Variations in recombination rates throughout the five chromosomes of *Arabidopsis* were recently tracked by constructing a high-resolution genetic map of single feature polymorphisms based on whole-genome array hybridization of recombinant inbred lines [58]. The average ratio of genetic to physical distance was 260 kb/cM genome-wide, with local differences ranging from 4 kb/cM to 3 Mb/cM. We ignored regional differences in recombination rates when establishing regression lines for map integration, in part because of uncertainties over the precise genetic locations of mapped visible markers. The use of locus numbers to track physical map distances was also subject to variation on a small scale. However, our success in identifying confirmed alleles based on estimated map locations demonstrates the utility of this approach for aligning genetic and physical maps on a large scale.

In some respects, the classical genetic map of *Arabidopsis*, which is limited to genes with mutant phenotypes mapped relative to each other, has outlived its usefulness as a reference for genetic analysis. The specific application described here represents a notable exception. Unlike other model organisms where the genetic map was constructed decades before genome sequencing was initiated and efficient methods of gene isolation were developed, the *Arabidopsis* classic map enjoyed a rather brief period of influence. Few genes have been added to the map in recent years and most new mutants are localized relative to molecular markers on the physical and recombinant inbred maps or recovered from T-DNA insertion lines amenable to rapid gene identification without mapping. Furthermore, the orders of many closely-linked genes on the classical map are inconsistent with their known physical locations, and there is no simple solution to this problem.

Several years ago, we published the first sequence-based map of genes with mutant phenotypes in *Arabidopsis*, which we concluded would eventually replace the classical genetic map [14]. We are currently updating that map based on information obtained from TAIR, NCBI, and the published literature. Ultimately, we hope to incorporate a comprehensive list of cloned genes with a loss-of-function phenotype into TAIR so that it can be widely used, thoroughly checked, and regularly updated by members of the community. The methods used here to compare genetic and physical map locations of genes with mutant phenotypes of interest should nevertheless continue to be relevant in future efforts to uncover the identities of mapped loci that remain to be cloned and in fully integrating the classical genetic literature of *Arabidopsis* [59] with ongoing research programs in functional genomics, natural variation, and systems biology.

## Materials and Methods

### Plant materials

Mutant alleles used in genetic complementation tests are listed in Table S4 (crosses that complemented) and Table S5 (crosses that failed to complement). Seeds for mapped and cloned *EMB* loci examined in our laboratory were obtained from internal stocks. Duplicates of these stocks are available through the *Arabidopsis* seed stock centers at Columbus, OH (ABRC) and Nottingham, UK (NASC). Several additional SALK [60], SAIL [61], and GABI [62] insertion lines were obtained from ABRC and NASC. The following stocks were obtained directly from other laboratories: *tps1* (Ian Graham, University of York, United Kingdom); *dom1, zeu1, cyl1, cyl2* (Martine Devic, CNRS-IRD-UP, Perpignan, France); *oep80* (Paul Jarvis, Leicester University, UK); *win1* (Jean Greenberg, University of Chicago, USA); and *pas2* (Jean-Denis Faure, INRA, Versailles, France). Seeds for *EMB3000* series mutants were obtained from Fumiyoshi Myouga and Kazuo Shinozaki (RIKEN Plant Science Center, Japan).

### Plant growth conditions

In order to track germination rates, observe segregating seedling phenotypes, and produce uniform populations of plants, we first plated mature seeds on a germination medium composed of Murashige and Skoog salts (MSP009, Caisson Laboratories, North Logan, UT USA), 3% glucose, and 0.8% agar. Seeds were surface-sterilized by a 30 sec exposure to 95% ethanol followed by a 6 min treatment with 50% Clorox (containing 1 drop of Tween 20 detergent per 10 ml), and then several rounds of washing with sterile water. Plates were stored in the refrigerator (4°C) for 2 days before being placed under fluorescent lights (16 hr light/8 hr dark cycles) at room temperature (23°C). After 14 to 17 days, seedlings were transplanted to pots containing a mixture of soil, sand, and vermiculite, watered daily with a nutrient solution, and maintained under long days in a plant growth room as described by Berg et al. [32].

### Genetic complementation tests

Desired heterozygotes were first identified by screening siliques from selfed plants for the presence of mutant seeds with the expected phenotype as explained in the tutorial section of the SeedGenes website (www.seedgenes.org/tutorial.html). Late floral buds with non-dehiscent anthers (female parent) were carefully emasculated with fine-tipped (Inox No. 4) forceps under a Wild (M7) dissecting microscope. A bent paper clip was often used to position the inflorescence on the microscope stage. Mature anthers from other heterozygous plants (male parent) were then applied to the stigma surface of the emasculated bud, covering it with pollen. Two or three open flowers immediately below the cross were removed to minimize pollen contamination. The number of flowers removed was recorded and each stem used for a cross was marked with colored thread for tracking purposes. Excess lateral branches were removed from the female parent to help direct nutrients to the inflorescence with the cross. The plant was then placed under fluorescent lights in a Percival (Perry, IA USA) plant growth chamber (AR-36L) and watered with the same nutrient solution used in the main growth room. We found that using a growth chamber improved the overall success rate of crosses performed, most likely due to increased humidity and light. The optimal time for screening each cross was dependent to some extent on the seed phenotype involved. However, most crosses were screened 10 days after the cross was performed. Complementation resulted in siliques with all normal seeds. Failure to complement resulted in siliques that continued to segregate for normal and mutant seeds. Three siliques were examined per inflorescence: the expected cross and one silique above and below. Typically, three sets of reciprocal crosses were performed for each cross combination to increase the reliability of results obtained.

### TAIL-PCR of plant sequences flanking T-DNA inserts

Five cauline leaves from heterozygous plants germinated on a selection medium containing 50 µg/ml kanamycin were collected and genomic DNA was extracted using a modified cetyl trimethyl ammonium bromide (CTAB) protocol [63]. Left border (LB) and right border (RB) T-DNA primers were used in various combinations with degenerate arbitrary primers or 10-mer random primers to amplify flanking genomic sequences. A complete list of primers used is presented in Table S6. TAIL-PCR reactions were performed with a Biometra (Göttingen, Germany) Uno II thermocycler using conditions described [6]. PCR products were separated in agarose gels, stained with ethidium bromide, and visualized with a Kodak (New Haven, CT USA) EDAS 290 camera. Amplified products were gel purified (Qiagen, Valencia, CA, USA) and sequenced at the Oklahoma State University Recombinant DNA/Protein Resource Facility. Pairs of gene-specific primers (GSP) were designed to flank each predicted insertion point based on BLAST results for TAIL-PCR amplicons. A confirmatory PCR with a GSP in combination with a T-DNA border primer (CMLB1 or CMLB2) was used to validate insert locations. PCR parameters employed were: 94°C for 2 min followed by 30 cycles of 94°C for 30 sec, 56°C for 40 sec, 72°C for 80 sec, and a final elongation step of 72°C for 5 min.

## Supporting Information

Table S1 Classical genetic map loci (excluding *EMB* genes) not cloned.
(0.02 MB XLS)

Table S2 Classical genetic map of *Arabidopsis* arranged by cM (all loci) or locus number (cloned genes).
(0.11 MB XLS)

Table S3 Estimated map locations of centromeric regions.
(0.01 MB XLS)

Table S4 Details on cross combinations that complemented (mutants not allelic).
(0.05 MB XLS)

Table S5 Details on cross combinations that failed to complement (mutants allelic).
(0.03 MB XLS)

Table S6 PCR primers used in the analysis of T-DNA insertion mutants.
(0.03 MB XLS)

Table S7 Locations of T-DNA inserts in mapped *emb* mutants.
(0.02 MB XLS)
